# Analgesic Effect and Potential Cumulative Benefit from Caudal Epidural D5W in Consecutive Participants with Chronic Low-Back and Buttock/Leg Pain

**DOI:** 10.1089/acm.2018.0085

**Published:** 2018-12-14

**Authors:** Liza Maniquis-Smigel, Kenneth Dean Reeves, Howard Jeffrey Rosen, John Lyftogt, Cassie Graham-Coleman, An-Lin Cheng, David Rabago

**Affiliations:** ^1^ Private Practice, Physical Medicine and Rehabilitation and Pain Management, Hilo and Honolulu, HI.; ^2^ Department of Physical Medicine and Rehabilitation, University of Kansas, Kansas City, KS.; ^3^ Private Practice, Anesthesiology and Pain Management, Anaheim and Monterey, CA.; ^4^ Private Practice, Retired, Christchurch, New Zealand.; ^5^ Registered Nurse, Kurtistown, HI.; ^6^ Department of Biomedical and Health Informatics, School of Medicine, University of Missouri-Kansas City, Kansas City, MO.; ^7^ Department of Family Medicine and Community Health, University of Wisconsin School of Medicine and Public Health, Madison, WI.

**Keywords:** epidural, dextrose, anesthesia caudal, low-back pain, neuralgia, neurogenic pain

## Abstract

***Objectives:*** Chronic low-back pain (CLBP) participants in a prior controlled study reported short-term pain relief after caudal epidural injection of 5% dextrose (D5W). This study assessed whether repeated caudal epidural injections of D5W results in serial short-term diminution of CLBP and progressive long-term decrease in pain and disability.

***Design:*** Prospective uncontrolled study.

***Settings/Location:*** Outpatient pain clinic.

***Subjects:*** Adults with CLBP with radiation to gluteal or leg areas.

***Interventions:*** Caudal epidural injection of 10 mL of D5W (without anesthetic) every 2 weeks for four treatments and then as needed for 1 year.

***Outcome measures:*** Numerical Rating Scale (NRS, pain, 0–10 points), Oswestry Disability Index (ODI, disability, %), and fraction of participants with ≥50% reduction in NRS score. Analysis by intention to treat.

***Results:*** Participants (*n* = 32, 55 ± 9.8 years old, nine female) had moderate-to-severe CLBP (6.5 ± 1.2 NRS points) for 11.1 ± 10.8 years. They received 5.5 ± 2.9 caudal D5W injections through 12 months of follow-up. The data capture rate for analysis was 94% at 12 months for NRS and ODI outcome measures, with 6% carried forward by intention to treat. A consistent pattern of analgesia was demonstrated after D5W injection. Compared with baseline status, NRS and ODI scores improved by 3.4 ± 2.3 (52%) and 18.2 ± 16.4% (42%) points, respectively. The fraction of participants with 50% reduction in NRS-based pain was 21/32 (66%).

***Conclusion:*** Epidural D5W injection, in the absence of anesthetic, resulted in consistent postinjection analgesia and clinically significant improvement in pain and disability through 12 months for most participants. The consistent pattern postinjection analgesia suggests a potential sensorineural effect of dextrose on neurogenic pain.

## Introduction

Nonsurgical chronic low-back pain (CLBP) is common, has high patient and societal impact, and is often refractory to best-practice care.^1^ With a global prevalence of 9.4%, it causes more disability than any other musculoskeletal condition.^2^ CLBP includes lumbar spinal stenosis, lumbar radiculopathy, failed back surgery, and nonspecific CLBP. Standard of care includes numerous therapies; none is uniformly effective. Harms associated with commonly used opioid prescription therapy are epidemic.^3^ Injection therapy is common nonsurgical care. Systematic reviews and trial reports have reported short-term efficacy of epidural injection of lidocaine with or without corticosteroid in spinal stenosis, radiculopathy,^4^ and failed back surgery.^5^ However, these studies have not confirmed long-term pain relief and systemic steroid effects can be problematic.^6^ Identification of effective and safe therapy for CLBP remains a public health priority.^7^

Dextrose in 12.5–25% concentration injection at entheses and intra-articular joint spaces for chronic musculoskeletal pain (prolotherapy) has been reported to reduce pain and improve function in a variety of conditions.^8,9^ A multifactorial mechanism has been proposed, including a direct sensorineural effect.^10^ Dextrose injections in 5–20% concentration have been used to treat superficial peripheral sensory nerves associated with chronic pain in uncontrolled^11–13^ and controlled studies.^14^

Dextrose 5–10% has also been safely injected into the epidural or intrathecal space to control epidural injectate placement.^15,16^ A recent single-injection double-blind study comparing the short-term analgesic effect of epidural 5% dextrose (D5W) with that of saline reported a safe and significant analgesic effect of D5W that endured for over 48 h.^17^ However, whether additional serial D5W injections would result in repeated short-term and enduring long-term pain diminution, and whether it has a concomitant effect on disability are not known. Therefore, we tested the hypothesis that serial caudal epidural injections of D5W result in serial short-term diminution of CLBP and a long-term decrease in pain and disability.

## Materials and Methods

### Recruitment criteria

The Western Institutional Review Board approved this study (ClinicalTrials.gov NCT01547364). The researchers recruited adults aged 19–75 from an outpatient physical medicine practice. Eligibility criteria were identical to those of the prior randomized controlled trial (RCT)^17^; inclusion criteria included nonsurgical back pain below the iliac crest for at least 6 months with accompanying buttock or leg pain ≥5 on a 0–10 point Numerical Rating Scale (NRS) in response to the question “What is the intensity of your pain?” and failure of physical therapy, massage therapy, or acupuncture. Exclusion criteria included pregnancy, progressive weakness, recent changes in opioid use, neurogenic bowel or bladder dysfunction, an unstable psychiatric disorder, local infection, current anticoagulation, or medical instability precluding study participation. All participants in both control saline and D5W arms of the prior RCT were offered enrollment in this open-label, nonrandomized prospective uncontrolled study.

### Diagnostic classification of participants

There is an absence of clear evidence-based guidelines for subgroup classification of CLBP with or without leg pain into tissue-specific diagnoses.^18^ Therefore, we assigned participants to CLBP subgroups based on two sets of criteria: (1) magnetic resonance imaging (MRI) or electromyographic (EMG) findings concordant with findings on clinical examination; and (2) consistency with recent clinical trials on efficacy of caudal epidural injection.^19,20^ These criteria resulted in five diagnoses. Lumbar spinal stenosis in the presence of pseudoclaudication plus moderate or severe MRI findings of stenosis;^21^ lumbar radiculopathy with both radicular symptoms and EMG findings consistent with radiculopathy;^22^ peripheral neuropathy with EMG findings of peripheral neuropathy;^23^ failed back surgery based on history, and non specific low back pain if examination, MRI, and EMG findings could not corroborate another diagnosis.

### Treatment pattern

Pilot studies by one of the co-authors (J.L.) utilized a weekly to biweekly injection frequency for peripheral perineural injection of dextrose 5–20% for neuropathic pain.^11–13^ Clinical observations after caudal epidural injection of D5W by two co-authors (L.M.-S. and H.J.R.) in patients with CLBP and buttock or leg pain suggested a rapid analgesic response and pain recurrence after 4–48 h. A durable response was generally observed after 3–4 biweekly injections (Unpublished data, L.M-S and H.J.R.), resulting in sustainable improvement. This study was continuous with the prior RCT: participants in the initial active arm received three additional biweekly D5W injections and participants in the initial control arm received four additional biweekly D5W injections. All participants were then offered additional caudal epidural D5W injections at 3, 6, and 9 months and by request. They were allowed to use oral analgesics if needed, but were encouraged to refrain from other interventional injections, physical therapy, or manipulation until 12 months.

### Injection description

Caudal epidural injections of 10 mL of D5W were administered at the level of the sacral hiatus, using a 25-gauge 3.8 cm needle, and a vertical needle entry, with confirmation of epidural injection by epidurography.^24^ Injections were performed by a fellowship-trained pain specialist (L.M.-S.) in an outpatient pain clinic.

### Outcome measures and diagnostic assessment

Baseline data collection occurred at the first caudal D5W injection. Pain was assessed using a single-item 0–10-point NRS in response to the question “What is the intensity of your back pain now?” at baseline, before, and following each injection (at 15 min in person and at 2, 4. and 48 h by telephone). The NRS score for pain is commonly used in studies of CLBP, including those assessing epidural injection.^19^ The minimal important change (MIC) in the NRS when assessing CLBP is 2.0 points or a 30% change from baseline.^25^

Back pain-specific functional impairment (“disability”) was assessed using the Oswestry Disability Index (ODI), a validated outcome for CLBP^26^ measured as a percentage from 0 to 100, with a higher percentage representing higher disability levels. Its MIC in CLBP is 10.0 or a 30% change from baseline.^25^

Long-term NRS and Oswestry scores were recorded in person or by telephone at 3, 6, 9, and 12 months after enrollment.

CLBP improvement was additionally assessed at 12 months by the percentage of participants who experienced a ≥50% reduction in NRS score, a measure commonly utilized in recent clinical trials to assess outcomes in low-back treatment, including injection therapy.^4,19^

Side effects, adverse events, and use of additional therapy were tracked. Demographics collected at baseline included age, sex, weight and height, medication intake, and specific CLBP diagnosis, and were used to characterize the sample and in covariate analyses.

### Statistical analysis

The data were analyzed by intention to treat using Predictive Analytics 180 software version 18.0.0 (PASW 18; IBM Corp., Armonk, NY). Descriptive statistics (mean ± standard deviation) were reported at baseline and at each time point for NRS pain and ODI values, and paired samples *t*-tests were used to analyze the change between baseline and each follow-up time point. Logistic regression analysis was utilized to determine if any baseline continuous variable (age, pain duration, pain improvement ≥50% at 15 min, and ODI value at baseline or body mass index [BMI]) or categorical variable (sex, diagnosis group, or narcotic intake) predicted long-term success (≥50% pain reduction at 12-month follow-up). Analysis of differences between different diagnostic groups was performed using multivariate analysis with Bonferroni correction for multiple groups.

## Results

### Study flow and demographics

**FIG. 1.**
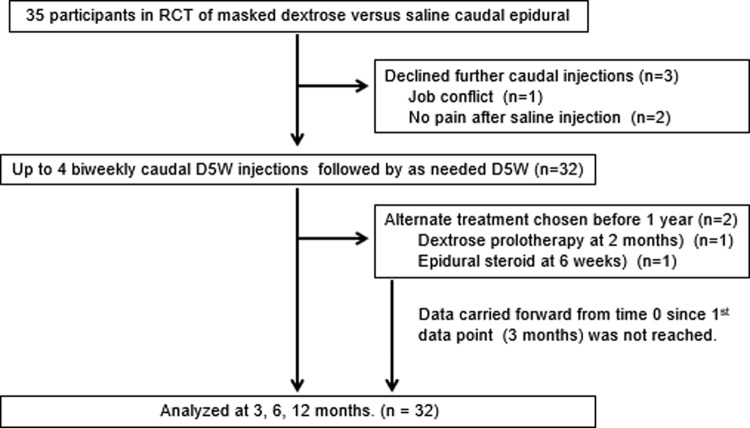
Enrollment and study flow. D5W, 5% dextrose; RCT, randomized controlled trial.

Biweekly injection of D5W was offered to all 35 participants who completed the prior RCT^17^ (Fig. 1). Three participants who received initial saline injection declined enrollment: two who had no residual pain and one with a job conflict. Thirty-two participants were enrolled beginning in February, 2012; the final data collection was in November, 2014. After initiation of treatment and before 3 months, two participants left the study to pursue nonprotocol therapy. Their data were carried forward from baseline for NRS and ODI. Data from all 32 participants were analyzed at 0, 3, 6, and 12 months. The participants (23 men and 9 women) had a mean age of 55 ± 9.8 years; 25 (78%) were either preobese (BMI ≥25–30 kg/m^2^) or obese (BMI ≥30 kg/m^2^) (Table 1). They had suffered from back pain for 11.1 ± 10.8 years (6.5 months to 50 years). Baseline pain severity was moderate to severe (6.5 ± 1.2 [4–9] points). Their mean ODI score was 43.5 ± 13.8%, which is in the lower range of severe disability (41–60).^27^ Seventeen participants (53%) were taking or had tried prescription of opioid medications for their low-back pain. The three most common diagnoses were lumbar spinal stenosis (*n* = 11, 34%), lumbar radiculopathy (*n* = 8, 25%), and nonspecific low-back pain (*n* = 7, 22%). The number of D5W injections through 3 months was 3.8 ± 1.2 and from 3 to 12 months was 1.8 ± 2.1.

**Table 1.** Baseline Demographics

*Characteristics*	
Female, *n* (%)	9 (28%)
Age, years, mean (SD)	55 ± 9.8
Pain duration, years, mean (SD)	11.1 ± 10.8
NRS pain, mean (SD)	6.5 ± 1.2
ODI 2.0, mean (SD)	43.5 ± 13.8
BMI, mean (SD)	30.1 ± 7.4
Opioid intake history, *n* (%)	17 (53%)
Medication history	
SSRI/SNRI intake, *n* (%)	3 (9%)
Gabapentin/pregabalin intake, *n* (%)	5 (16%)
Steroid epidural, *n* (%)	7 (22%)
Diagnosis	
Lumbar spinal stenosis, *n* (%)	11 (34%)
Lumbar radiculopathy, *n* (%)	8 (25%)
Nonspecific low-back pain, *n* (%)	7 (22%)
Failed back surgery, *n* (%)	4 (13%)
Peripheral neuropathy, *n* (%)	2 (6%)

BMI, body mass index; NRS, Numerical Rating Scale; ODI, Oswestry Disability Index; SD, standard deviation; SSRI, selective serotonin reuptake inhibitor; SNRI, serotonin and norepinephrine reuptake inhibitor; medication intake percentages include both current and past usage.

### Short-term outcomes

**FIG. 2.**
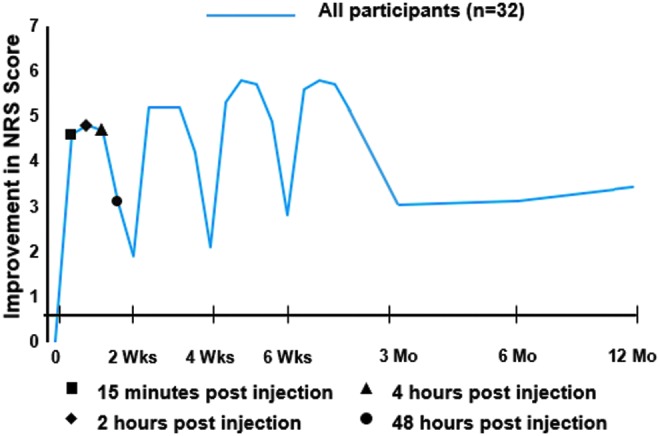
Analgesic response to 5% dextrose caudal epidural injection and long-term pain course. NRS, Numerical Rating Scale.

NRS pain change scores after each of the initial biweekly injections were distributed in consistent reverse-U-shaped curves with diminution of pain ≥70% compared with preinjection scores at 15 min, and 2 and 4 h (*p* < 0.001), with continued significant improvement through 48 h (*p* < 0.05) (Fig. 2). Longer-term cumulative benefit was suggested by the step-wise decrease in preinjection and postinjection pain score with each injection.

### Twelve-month outcomes

**FIG. 3.**
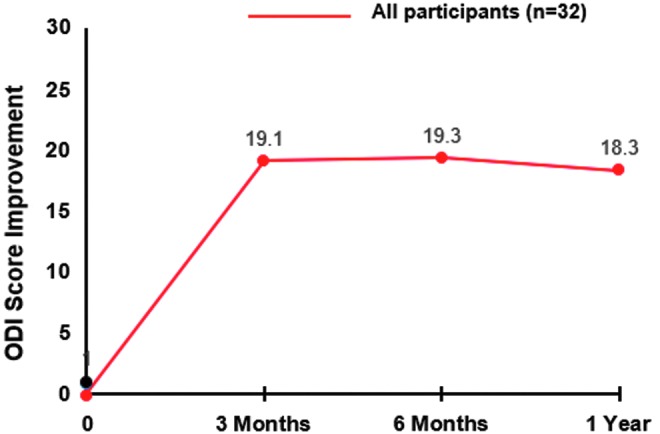
Improvement in ODI score over 1 year. ODI, Oswestry Disability Index.

In long-term follow-up, participants (*n* = 32) reported improvement in NRS pain and ODI scores compared to baseline status (Table 2). Most of the improvement was attained by 3 months and sustained through 12 months (Figs. 2 and 3), at which time, improvement in the NRS pain score was 3.4 ± 2.3 points (52%; *p* < 0.001) and improvement in the ODI score was 18.3% ± 16.4% (42%; *p* < 0.001), exceeding 1.5 times the MIC for improvement in NRS and ODI scores in patients with low-back pain.^25^ Sixty-six percent (21/32) of participants achieved a ≥50% improvement in NRS pain score. There were no significant predictors of ≥50% improvement at 12 months. The data capture rate for analysis was 94% at 12 months for NRS and ODI outcome measures.

**Table 2.** Raw Score and Change Scores for Numerical Rating Scale and Oswestry Disability Index over Time

	*Baseline*	*3 Months*	*6 Months*	*12 Months*
	n* = 32*	n* = 32*	n* = 32*	n* = 32*
Raw score (SD)	6.5 (1.2)	3.5 (2.3)	3.4 (2.2)	3.1 (2.3)
Change (SD)	NA	3.0 (2.3)^a^	3.1 (2.2)^a^	3.4 (2.3)^a^

	*Baseline*	*3 Months*	*6 Months*	*12 Months*
Raw score (SD)	43.5 (13.8)	24.5 (15.6)	24.3 (15.8)	25.3 (16.9)
Change (SD)	NA	19.1 (14.1)^b^	19.3 (14.2)^b^	18.3 (16.4)^b^

^a^ Pain values were significantly different from baseline at 3 (*p* < 0.001), 6 (*p* < 0.001) and 12 months (*p* < 0.001).

^b^ ODI values were significantly different from baseline at 3 (*p* < 0.001), 6 (*p* < 0.001) and 12 months (*p* < 0.001).

NA, not applicable; ODI, Oswestry Disability Index; SD, standard deviation.

**FIG. 4.**
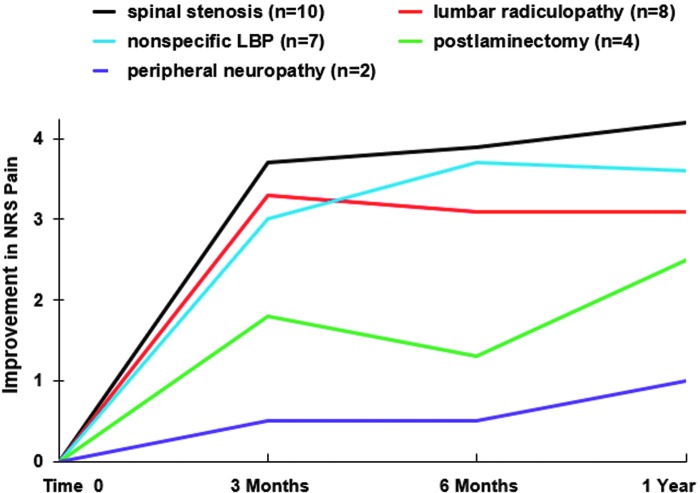
Improvement in NRS pain score over time by diagnostic group. LBP, low-back pain; NRS, Numerical Rating Scale.

**FIG. 5.**
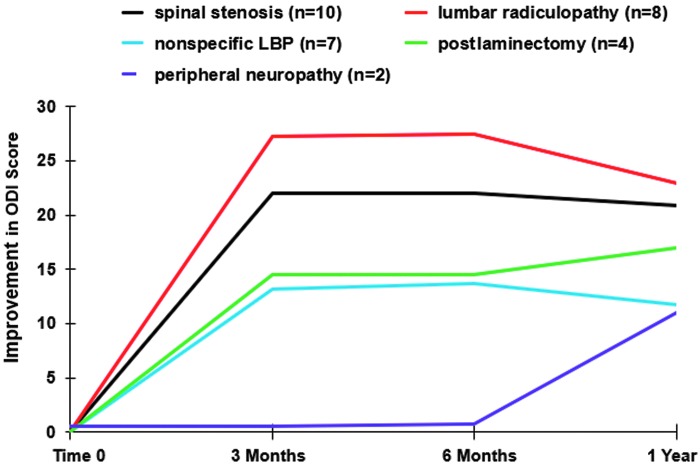
Improvement in ODI score over time by diagnostic group. LBP, low-back pain; ODI, Oswestry Disability Index.

NRS and ODI change scores varied by diagnostic group through 12 months (Table 3). Participants with spinal stenosis, nonspecific low-back pain or lumbar radiculopathy responded well, with significant NRS score changes of 4.2 ± 2.2 (*p* < 0.001), 3.0 ± 2.6 (*p* = 0.01), and 3.6 ± 2.3 (*p* = 0.006) points, respectively, and ODI percentage changes of 20.9 ± 18.1 (*p* = 0.003), 22.9 ± 23.9 (*p* = 0.03), and 11.7 ± 6.7 (*p* = 0.004) (Figs. 3–5). Failed back surgery participants reported significant ODI improvement (17.0 ± 6.8 [*p* = 0.02]). Peripheral neuropathy participants reported no significant change. There were no between-group differences (*p* = 0.179).

**Table 3.** Raw Score and Change Scores for Numerical Rating Scale and Oswestry Disability Index Over Time by Diagnostic Category

*Group*	*Measure*	*Baseline*	*3 Months*	*6 Months*	*12 Months*
NRS 0–10					
Spinal stenosis	Raw score (SD)	6.6 (1.3)	2.9 (1.6)	2.7 (1.4)	2.5 (1.6)
*n* = 11	Change (SD)	NA	3.7 (2.5)^a^	3.9 (2.2)^a^	4.2 (2.2)^a^
Lumbar radic.	Raw score (SD)	5.5 (1.1)	2.4 (2.4)	2.5 (2.1)	2.5 (3.1)
*n* = 8	Change (SD)	NA	3.3 (2.1)^b^	3.0 (1.9)^b^	3.0 (2.6)^b^
Nonspecific LBP	Raw score (SD)	6.6 (1.1)	3.6 (1.6)	2.9 (1.3)	3.0 (1.5)
*n* = 7	Change (SD)	NA	3.0 (2.4)^c^	3.7 (2.1)^d^	3.6 (2.3)^d^
Postlaminectomy	Raw score (SD)	6.8 (0.5)	5.0 (2.3)	5.5 (1.9)	4.3 (1.7)
*n* = 4	Change (SD)	NA	1.8 (2.1)	1.3 (1.5)	2.5 (1.7)
Periph. neurop.	Raw score (SD)	8.5 (0.7)	8.0 (0.0)	8.0 (0.0)	7.5 (0.7)
*n* = 2	Change (SD)	NA	0.5 (0.7)	0.5 (0.7)	1.0 (1.4)
ODI (0–100)					
Spinal stenosis	Raw score (SD)	45.4 (17.0)	23.4 (15.2)	23.4 (15.2)	24.5 (13.4)
*n* = 11	Change (SD)	NA	22.0 (14.2)^c^	22.0 (14.2)^c^	20.9 (18.1)^c^
Lumbar radic.	Raw score (SD)	43.3 (14.5)	16.0 (17.2)	15.8 (17.7)	20.4 (27.8)
*n* = 8	Change (SD)	NA	27.3 (15.6)^e^	27.5 (16.3)^e^	22.9 (23.9)^e^
Nonspecific LBP	Raw score (SD)	38.3 (9.9)	25.1 (10.2)	24.6 (9.9)	26.6 (8.5)
*n* = 7	Change (SD)	NA	13.2 (7.8)^f^	13.7 (7.7)^f^	11.7 (6.7)^f^
Postlaminectomy	Raw score (SD)	45.0 (12.5)	30.5 (10.9)	30.5 (10.9)	28.0 (8.2)
*n* = 4	Change (SD)	NA	14.5 (10.9)	14.5 (10.9)	17.0 (6.8)^g^
Periph. neurop.	Raw score (SD)	50.0 (11.3)	50.0 (11.3)	50.0 (11.3)	39.0 (12.8)
*n* = 2	Change (SD)	NA	0.0 (0.0)	0.0 (0.0)	11.0 (1.4)

^a^ Pain values were significantly different from baseline at 3 (*p* = 0.001), 6 (*p* < 0.001), and 12 months (*p* < 0.001).

^b^ Pain values were significantly different from baseline at 3 (*p* = 0.003), 6 (*p* = 0.002), and 12 months (*p* = 0.01).

^c^ ODI values were significantly different from baseline at 3 (*p* < 0.001), 6 (*p* < 0.001), and 12 months (*p* = 0.003).

^d^ Pain values were significantly different from baseline at 3 (*p* = 0.016), 6 (*p* = 0.003), and 12 months (*p* = 0.006).

^e^ ODI values were significantly different from baseline at 3 (*p* = 0.002), 6 (*p* = 0.002), and 12 months (*p* = 0.03).

^f^ ODI values were significantly different from baseline at 3 (*p* = 0.004), 6 (*p* = 0.003), and 12 months (*p* = 0.004).

^g^ ODI values were significantly different from baseline at 12 months (*p* = 0.02).

LBP, low-back pain; Lumbar Radic., lumbar radiculopathy; NA, not applicable; NRS, Numerical Rating Scale; ODI, Oswestry Disability Index; Periph. Neurop., peripheral neuropathy; SD, standard deviation.

One hundred and ninety-two caudal epidural injections were performed during the 12-month treatment period. No vasovagal events, postprocedure discomfort, or other adverse effects were reported. Anecdotally assessed subject satisfaction was high.

## Discussion

This open-label study of serial caudal epidural D5W injections using a small-needle vertical technique^24^ in patients with one of several different CLBP diagnoses had two main findings. First, participants reported consistent analgesia after each injection, which peaked within 15 min and lasted 48 h. Second, participants in all diagnostic groups, except peripheral neuropathy, reported clinically important and statistically significant improvements in pain and/or function at 12 months.

The finding that NRS scores improved in the first 48 h after injection is consistent with our prior work.^17^ D5W-injected participants in the prior RCT improved substantially when compared to saline controls through 48 h, suggesting that D5W has biological activity in this context. This study adds the findings that D5W confers a consistent pattern of short-term pain reduction after each of several D5W injections over time, and that after each D5W injection, the postinjection pain peak after 48 h is less than the preinjection pain, with progressive diminution of pain through 12 months.

A prior RCT has reported the independent clinical effect of D5W compared with blinded saline injections for chronic pain.^8^ The mechanism of action of D5W injection in acute and chronic pain is unclear and has been hypothesized to be multifactorial.^10,28^ Traditional prolotherapy, which includes injection of 12.5–25% dextrose within joint spaces and at bony soft tissue attachments,^8^ is thought to initiate a tissue-level inflammatory response favoring anabolic processes,^29,30^ which is unlikely to be the mechanism in this study, given that D5W is low concentration and does not have an inflammatory effect.^31,32^

What cellular mechanisms may explain the apparent analgesic effect of D5W in the prior RCT and in this longer-term study? One randomized controlled study suggests a potential nerve-specific, or sensorineural, mechanism for pain reduction following perineural injection of D5W.^33^ The literature suggests three potential sensorineural mechanisms. First, dextrose may act at the level of a key pain modulator, for example, an ion channel. The transient receptor potential vanilloid receptor-1 (TRPV1) ion channel plays a central role in the development of allodynia and hyperalgesia in patients with chronic pain.^34,35^ Chronic neuropathic pain is associated with persistent upregulation of the TRPV1 ion channel.^36^ Mannitol, a metabolically inert sugar molecule that is structurally similar to dextrose, has been reported to reduce pain resulting from upregulation of TRPV1 ion channels in an RCT using a capsaicin pain model.^37^ Perineural injection of D5W and 5% mannitol have been anecdotally observed to have similar effects on pain in a co-author's clinic (J.L.). Although TRPV1 ion channels have no monosugar receptors,^35^ certain monosugars may modulate the effects of TRPV1 expression in an allosteric manner^35^ through a class effect.

Second, dextrose may replenish low energy stores in the context of chronic pain. Peripheral nerves are particularly sensitive to glycopenia,^38,39^ and develop histopathologic evidence of damage with repeated reduction of systemic dextrose by only 25%.^39^ Perineural glycopenia results in progressive depolarization and hyperexcitability of nociceptive nerve fibers, presumably through reduced effectiveness of the ATPase pump, which depends on dextrose for ATP production.^40^ In one study, nociceptive C-fibers exposed to a temporary glycopenic environment demonstrated a 653% ± 23% increase in action potential frequency within 15 min, with prompt return to a normal firing rate upon return to baseline dextrose levels.^40^ Dextrose injections may provide analgesia through correction of local glycopenia. However, confirming that the perineural environment is relatively glycopenic will require microdialysis or other analysis methods for confirmation.^41^

Third, elevation of extracellular dextrose levels by dextrose injection may hyperpolarize nerves through another mechanism. For example, activation of tandem-pore K+ channels by dextrose leads to increased K+ conductance and resulting neuronal hyperpolarization.^42^ Elevation of extracellular dextrose to 0.5% from normal levels of 0.1% in the gut hyperpolarizes enterocyte cell membranes promptly by the Na/dextrose cotransporter (SGLT1),^43^ but SGLT1 has a much less significant role in transport across neuronal membranes.^44^ Although the mechanism of nociceptive fiber calming by dextrose injection has not been confirmed, a hyperpolarization effect is consistent with recent reports of co-administration of D5W to decrease the pain from infusion of certain chemotherapeutic agents^45,46^ or microspheres.^47^

In this study, CLBP may be partially a product of sensitization of small fibers in the somatosensory system, particularly C-fibers expressing a TRPV1 channel, which is increasingly recognized as an important sensor ion channel both within and peripheral to the spinal cord.^36,48^ The source of nociceptive C-fiber sensitization is likely to be multifactorial, given the diversity of participants with low-back pain enrolled in this study. The injection of epidural D5W may reduce the firing threshold of nociceptive C-fibers by one or more of the mechanisms described above. However, these potential mechanisms, while informed by the medical literature, are speculative, have not been formally tested, and do not explain the temporal effect of diminishing pain with serial injections.

Although this study did not utilize hypertonic (> 6.5%) levels of dextrose, the cumulative long-term effect of serial epidural D5W injections on pain and dysfunction may provide a partial explanation for the favorable outcomes seen in RCTs assessing hypertonic dextrose injection for the treatment of chronic painful musculoskeletal conditions such as knee osteoarthritis,^8,49–51^ Osgood Schlatter disease,^52^ rotator cuff tendinopathy,^53^ hand osteoarthritis,^54,55^ Sacroiliac joint dysfunction,^56^ and lateral epicondylosis.^57^ Longer-term pain diminution in this study is also consistent with case series and randomized controlled studies assessing subcutaneous dextrose 5–20% injection for pain syndromes with a neuropathic pain component.^11–14^

The limitations of this study include a small sample size and lack of a control group. In addition, control participants received epidural saline as part of the preceding RCT and then received epidural D5W without a washout period, potentially affecting the results, although the effect of epidural saline was minimal.^17^ This study was not powered to detect rare events, but caudal epidural D5W injection appeared to be safe; no unexpected side effects or adverse effects were reported. This is consistent with a lack of evidence of toxicity of dextrose in multiple previous studies, in which dextrose 5–10% was included in anesthetic solutions injected in the subdural space to facilitate distribution of the injectate.^15,16^ The strengths of this study include generalized low-back pain as an eligibility criterion, which facilitated the inclusion of several different nonsurgical subgroups. Participant retention and data collection were effective. Further studies are indicated to better evaluate the clinical indications and effects of epidural D5W compared both to control and active therapy. While the safety of D5W is advantageous, future work should also consider other concentrations of dextrose, and be powered to detect rare procedure-related adverse events.

## Conclusion

Serial caudal epidural injection of D5W resulted in rapid, serial short-term analgesia and a progressive decrease in pain and disability through 12 months among participants with CLBP with buttock and leg pain of varying etiologies. Caudal D5W injections may be appropriate therapy for some patients with CLBP.
